# Reference Gene Selection for qRT-PCR Analysis in the Sweetpotato Whitefly, *Bemisia tabaci* (Hemiptera: Aleyrodidae)

**DOI:** 10.1371/journal.pone.0053006

**Published:** 2013-01-08

**Authors:** Rumei Li, Wen Xie, Shaoli Wang, Qingjun Wu, Nina Yang, Xin Yang, Huipeng Pan, Xiaomao Zhou, Lianyang Bai, Baoyun Xu, Xuguo Zhou, Youjun Zhang

**Affiliations:** 1 Institute of Pesticide, Hunan Agricultural University, Changsha, P. R. China; 2 Department of Plant Protection, Institute of Vegetables and Flowers, Chinese Academy of Agricultural Sciences, Beijing, P. R. China; 3 Department of Entomology, University of Kentucky, Lexington, Kentucky, United States of America; East Carolina University, United States of America

## Abstract

**Background:**

Accurate evaluation of gene expression requires normalization relative to the expression of reliable reference genes. Expression levels of “classical” reference genes can differ, however, across experimental conditions. Although quantitative real-time PCR (qRT-PCR) has been used extensively to decipher gene function in the sweetpotato whitefly *Bemisia tabaci*, a world-wide pest in many agricultural systems, the stability of its reference genes has rarely been validated.

**Results:**

In this study, 15 candidate reference genes from *B. tabaci* were evaluated using two Excel-based algorithms *geNorm* and *Normfinder* under a diverse set of biotic and abiotic conditions. At least two reference genes were selected to normalize gene expressions in *B. tabaci* under experimental conditions. Specifically, for biotic conditions including host plant, acquisition of a plant virus, developmental stage, tissue (body region of the adult), and whitefly biotype, *ribosomal protein L29* was the most stable reference gene. In contrast, the expression of *elongation factor 1 alpha*, *peptidylprolyl isomerase A*, *NADH dehydrogenase*, *succinate dehydrogenase complex subunit A* and *heat shock protein 40* were consistently stable across various abiotic conditions including photoperiod, temperature, and insecticide susceptibility.

**Conclusion:**

Our finding is the first step toward establishing a standardized quantitative real-time PCR procedure following the MIQE (Minimum Information for publication of Quantitative real time PCR Experiments) guideline in an agriculturally important insect pest, and provides a solid foundation for future RNA interference based functional study in *B. tabaci*.

## Introduction

In recent years, quantitative real-time PCR (qRT-PCR) has been widely utilized for gene expression analysis because of its sensitivity, accuracy, reproducibility, and most importantly, quantitativeness [1]–[4]. There is no argument that qRT-PCR is a powerful tool for gene expression analysis, data analysis, and subsequent interpretation. However, interpretation can be challenging due to variation caused by pipetting error and different extraction techniques, transcription and amplification efficiencies among different samples [2], [5]–[7]. Therefore, controlling for internal differences and reducing errors between samples requires the use of reliable reference genes for normalization in gene expression analysis [8].

Traditionally, housekeeping genes including 18S ribosomal RNA, glyceraldehyde-3-phosphate dehydrogenase, elongation factor, ubiquitin-conjugating enzyme, alpha microtubules protein, and beta microtubule protein have been used extensively as endogenous controls for the normalization of qRT-PCR data, but the expression levels of these reference genes can differ under environmental conditions [9]. Based on previous studies, it is evident that the existence of a single universal reference gene suited for all experimental conditions is highly unlikely [5], [10]–[12]. Therefore, selection of reliable reference genes that are consistently expressed under specific experimental conditions is critical for the interpretation of qRT-PCR results. Currently, two Excel-based software tools including *geNorm* (http://medgen.ugent.be/~jvdesomp/genorm/) [10] and *Normfinder* (http://www.mdl.dk/publications *normfinder*.htm) [13], are widely used for evaluating the performance of reference genes. The *geNorm* program was used to calculate the mean pair-wise variation between an individual gene and all other tested candidate reference genes and the results were shown as expression stability (M). *Normfinder* is an algorithm for estimation of reference genes among a set of candidates. It ranks the candidate genes based on their expression stability.

The sweetpotato whitefly *Bemisia tabaci* (Gennadius) (Hemiptera: Aleyrodidae) is one of the most destructive insect pests worldwide [14]–[15]. This whitefly damages many crops by direct feeding and by vectoring 114 plant viruses [16]. *Bemisia tabaci* has long been thought to comprise morphologically indistinguishable biotypes that often differ in host range, fecundity, insecticide resistance, transmission competency for begomoviruses, and the symbionts they harbor [14], [16], [17]. Recent studies suggest that most of these biotypes represent genetically distinct cryptic species [18]–[19], among which the B biotype of the Middle East-Minor Asia 1 and the Q biotype of the Mediterranean group are the most invasive and destructive [20]. Although *B. tabaci* was first recorded in China in the late 1940s, crop damage caused by this insect did not become serious until the introduction of the B biotype in the 1990s [21]. The Q biotype of *B. tabaci* was first detected in Yunnan Province, China in 2003 [22]. Since then, the Q biotype has gradually displaced the established B populations and has become the dominant *B. tabaci* in most of China [23].

To examine the temporal and spatial changes of gene expression in *B. tabaci*, *β-actin* and *α-tubulin* are the most frequently used endogenous reference genes in qRT-PCR analyses [24]–[27]. These genes were selected without the companion validation study to evaluate their suitability under specific experimental conditions. Previous studies have demonstrated that the expression of *β-actin* can be significantly influenced by tissue type [12]. Bustin and his colleagues proposed a MIQE guideline (Minimum Information for publication of Quantitative real time PCR Experiments) [28] to standardize qRT-PCR analysis; reference gene selection is an integral part of their recommendations. In this study, 15 housekeeping genes from a parallel *B. tabaci* transcriptome study [29] were selected as candidate reference genes. The overall goal of this research is to develop a standardized qRT-PCR analysis in *B. tabaci* following the MIQE guideline. Specifically, we evaluate the stability and performance of the above mentioned candidate reference genes under different experimental conditions including five biotic factors (host, acquisition of a plant virus, developmental stage, tissue, and whitefly biotype) and three abiotic factors (photoperiod, temperature, and insecticide exposure). The choice and number of reference genes needed under various conditions are investigated and recommended.

## Materials and Methods

### Ethics Statement

*Bemisia tabaci* B biotype strains used in this study were initially collected in the field at Beijing in 2000, and have been maintained in a greenhouse at the Institute of Vegetables and Flowers, Chinese Academy of Agricultural Sciences. The species in the genus Aleyrodidaeare common agricultural pests and are not included in the “List of Protected Animals in China”. No specific permits were required for the described field studies.

### Candidate Reference Genes

Housekeeping genes from a previous *B. tabaci* transcriptomic study [29] were selected as candidate reference genes including *β-actin* (*Actin*), *18S rRNA* (*18S*), *heat shock protein* (*HSP20*, *HSP40*, *HSP70*, *HSP90*), *γ-tubulin*, *60S ribosomal protein L29* (*RPL29*), *succinate dehydrogenase complex subunit A* (*SDHA*), *flavoprotein*, *glyceraldehyde phosphate dehydrogenase* (*GAPDH*), *elongation factor 1 alpha* (*EF-1α*), *peptidylprolyl isomeraseA* (*PPIA*), *NADH dehydrogenase* (*NADH*), *Myosin light chain* (*Myosin L*), and *adenosine triphosphate enzyme* (*ATPase*). Primer 5.0 (http://www.premierbiosoft.com/) was used to design primers for qRT-PCR analysis. The validity of these candidate reference genes were evaluated under selected biotic and abiotic conditions described in the following sections.

### Biotic Conditions

#### Host plant

*Bemisia tabaci* B biotype was maintained on three different host plants including cabbage, tomato, and cucumber [30]. A total of 180 3-day-old adults were collected, snap frozen in liquid nitrogen, and stored at −80°C before qRT-PCR analysis.

#### Acquisition of a plant virus

Tomato plants infected with Tomato yellow leaf curl virus (TYLCV) were obtained by *Agrobacterium tumefaciens*-mediated inoculation using a cloned TYLCV genome (GenBank accession ID: AM282874) [31]. Plants were inoculated with the virus at the 3-true-leaf stage. Viral infection of tomato plants was confirmed by the development of characteristic leaf curl symptoms and was further validated by molecular analysis [32]. Viruliferous *B. tabaci* were obtained by caging non-viruliferous *B. tabaci* adults with TYLCV-infected tomato plant for a 72 h acquisition access period [33]. Non-viruliferous *B. tabaci* were obtained by caging non-viruliferous *B. tabaci* adults with healthy tomato plants for 72 h. A total of 180 3-day-old adult whiteflies from both virus-infected and virus-free tomato plants, respectively, were snap frozen and stored as described earlier.

#### Developmental stage

Three developmental stages (egg, pupa, and adult) were collected from *B. tabaci* B biotype maintained on healthy cabbage plants. A total of 900 eggs, 900 pupae, and 300 adults were snap frozen and stored as described earlier.

#### Tissue

A dissection needle and a stereo microscope (Leica, DFC425) were used to obtain three body regions (head, thorax, and abdomen) from 3-day-old *B. tabaci* adults (TH-S). These sections were dissected from adults reared on cabbage plants; snap frozen, and stored as described earlier.

#### Whitefly biotype

*Bemisia tabaci* B and Q biotype strains were collected from Beijing, China in 2000 and 2008, respectively, and have been maintained in a greenhouse at the Institute of Vegetables and Flowers, Chinese Academy of Agricultural Sciences [30].

### Abiotic Conditions

#### Photoperiod

A total of 200 3-day-old *B. tabaci* adults were placed into nine screen cages, respectively, and provisioned with cabbage plants at the 5 to 7-true-leaf stage. These cages were kept in growth chambers (27±0.5°C, 60±10% RH) with photoperiods (L/D) of 24∶0, 0∶24, and 14∶10, respectively. After 96 h, *B. tabaci* adults were snap frozen and stored as described earlier.

#### Temperature

A total of 720 3-day-old *B. tabaci* adults (80 whiteflies×9 replications) were collected from cabbage plant and placed individually into 30 ml specimen tubes. The tubes were then placed in climatic chambers at 4.0, 25.0, and 37.5°C, respectively. After 1 h, the live adults were snap frozen and stored as described earlier.

#### Insecticide susceptibility

Thiamethoxam susceptible (TH-S) and resistant (TH-R) *B. tabaci* strains were established from the same populations described previously [34]. Before sample collection, a leaf-dip bioassay [34] was conducted to confirm that the resistance factor [LC50 (TH-R)/LC50 (TH-S)] was over 70-fold. A total of 180 adults from both TH-S and TH-R were collected, snap frozen, and stored as described earlier.

### Total RNA Extraction and cDNA Synthesis

Total RNA was extracted with a Trizol reagent (Invitrogen, Carlsbad, CA, USA). RNA was quantified by measuring the absorbance at 260 nm with a Nano Vue UV/Vis spectrophotometer (GE Healthcare). The purity of RNA was assessed at an absorbance ratio of OD260/280 and OD260/230, and the integrity was checked with 1% agarose gel electrophoresis. Then, 1 µg of RNA was used to synthesize the first-strand cDNA using the PrimeScript®RT reagent Kit (Takara Bio, Tokyo, Japan) with gDNA Eraser (Perfect Real Time, TaKara, Shiga, Japan) according to the manufacturer's protocol. The synthesized cDNA was stored at −20°C.

### Quantitative Real-time PCR analysis

Quantitative Real-time PCR (qRT-PCR) was performed on an ABI 7500 real-time system. The cDNA of each sample representing one biological replicate was diluted to a working concentration of 17 ng/µl for the qRT-PCR analysis. The melt temperature was 60°C and product contained between 80 and 200 base pairs (Table 1). The 25 µl reaction system contained 1 µl of diluted cDNA, 11.25 µl of SYBR® Green Real-time PCR Master Mix (TIANGEN, Corp, Beijing, China), and 0.5 µl of each primer. The cycling parameters were as follows: 95°C for 3 min followed by 40 cycles of 95°C for 30 s, 60°C for 30 s, and 72°C for 35 s. A 3-fold serial dilution of cDNA was used to construct a standard curve to determine the PCR efficiency that would be used to convert the quantification cycles (Ct-values) into the relative quantities (relative gene expressions).

**Table 1:** Primers used for qRT-PCR analysis.

Gene	Accession Number	Primer sequences (5′to 3′)^1^	Amplicon (bp)	Tm (°C)^2^	E (%)^3^	*R* 2 4
*HSP20*	EU934239	F-AAGAAGTCAGCGTGAAAGTCGR-GTACCTCCTAGTGAAAGATCGG	107	60	99.5	0.9978
*HSP40*	EE597535	F-AGATGAGGCTCATGATGGTCAAR-TGAGAAGCGCATTGCATTGT	81	60	109.4	0.9992
*HSP70*	EU934240	F-AGCACTCCGGCGTCTACGR-CGAACCTGGCACGGGACAC	134	60	109.6	0.9944
*HSP90*	EU934241	F-ATCGCCAAATCTGGAACTAAAGCR-GTGTTTTGAGACGACTGTGACGGTG	135	60	100.9	0.9951
*PPIA*	JU470456	F-ATGTTTTGGGCTTTGGTCR-CGTTGCCATCTGAATGAAATAC	148	60	96.9	0.9988
*EF-1α*	EE600682	F-TAGCCTTGTGCCAATTTCCGR-CCTTCAGCATTACCGTCC	110	60	103.9	0.998
*SDHA*	JU470457	F-GCGACTGATTCTTCTCCTGCR-TGGTGCCAACAGATTAGGTGC	141	60	92.4	0.9986
*NADH*	JU470455	F-ATAGTTGGCTGTAGAACCAGAGTGR-ACACGAAGGGAAGAGCACATA	96	60	93.5	0.9973
*γ- tubulin*	JU470458	F-CCACAATCCATGCAAATCR-CCGAAATGGCCTCTGCTA	117	60	75.3	0.9832
*Myosin L*	EE597481	F-TTTCAGACGAGGATGTCGCAR-CGTCATAGATTTCGAACGCG	81	60	108.0	1.0000
*RPL29*	EE596314	F-TCGGAAAATTACCGTGAGR-GAACTTGTGATCTACTCCTCTCGTG	144	60	101.3	0.9909
*ATPase*	JU470453	F-AGAGCGAGTGTTTGGGTGR-GACGGCGATTCGAGAAGG	138	60	98.9	0.9994
*18S*	U20401	F-CGGCTACCACATCCAAGGAAR-GCTGGAATTACCGCGGCT	187	60	99.5	0.9987
*Actin*	AF071908	F-TCTTCCAGCCATCCTTCTTGR-CGGTGATTTCCTTCTGCATT	174	60	95.0	0.9973
*GAPDH*	JU470454	F-GGACACGGAAAGCCATACCAGR-ACCACCGCTACCCAAAAGACC	166	60	77.0	0.9943

“1” : F, forward primer; R, reverse primer;

“2” : Tm, Annealing temperature;

“3” : E, Efficiency;

“4” : *R* ^2^, Coefficient of determination.

### Data Analysis

Expression of reference genes was evaluated with two web-based analysis tools: *geNorm* and *NormFinder*. *geNorm* was used to calculate the *M* stability value as the mean pairwise variation between an individual gene and all other tested candidate genes. The lower the *M* value, the more stable the reference genes. The value of V_n_/V_n+1_ indicates the pairwise variation between two sequential normalization factors and determines the optimal number of reference genes required for accurate normalization. A value below 0.15 indicates that an additional reference gene will not significantly improve normalization. *Normfinder* evaluates the overall variation of the candidate reference genes under the experimental conditions and estimates the variation between and within groups. For each candidate gene, *Normfinder* provides a stability value that is a direct and rapid measurement of expression variation. This stability value enables the user to estimate the systematic error introduced when selecting a suitable reference gene.

## Results

### Expression profiles of candidate reference genes

For each reference gene, a dissociation curve with a single-peak ensured that the primer sets amplified a unique PCR product ranging from 81 to 187 bp. The PCR efficiency was consistently high for candidate reference genes except *γ-tubulin* (75.3%) and *GAPDH* (77.0%) (Table 1). The raw Ct values ranged from 11.63 (*18S*) to 31.11 (*γ-tubulin*) with different host plants; from 12.02 (*18S*) to 31.25 (*HSP70*) with different photoperiods; from 11.85 (*18S*) to 30.37 (*γ-tubulin*) with different temperatures; from 11.49 (*18S*) to 30.86 (*HSP70*) for non-viruliferous and viruliferous adults; from 12.44 (*18S*) to 29.58 (*γ-tubulin*) in thiamethoxam-resistant and -susceptible adults; from 12.17 (*18S*) to 29.58 (*γ-tubulin*) for different developmental stages; from 9.15 (*18S*) to 33.13 (*γ-tubulin*) for different tissues; and from 12.56 (*18S*) to 30.71 (*γ-tubulin*) for B and Q biotype adults.

### Stability of candidate reference gene expression

#### *geNorm*

The *geNorm* program was used to calculate the average expression stability values (*M* stability values) and to plot the influence of different factors using pairwise comparisons. The least stable genes have the highest *M* values and were successively excluded. The program also indicated the minimum number of reference genes for accurate normalization in *B. tabaci* by the pairwise variation value. Values (V2/3) under 0.15 shows that no additional genes are required for the normalization (Figure S1 and S2).

**Figure 1:**
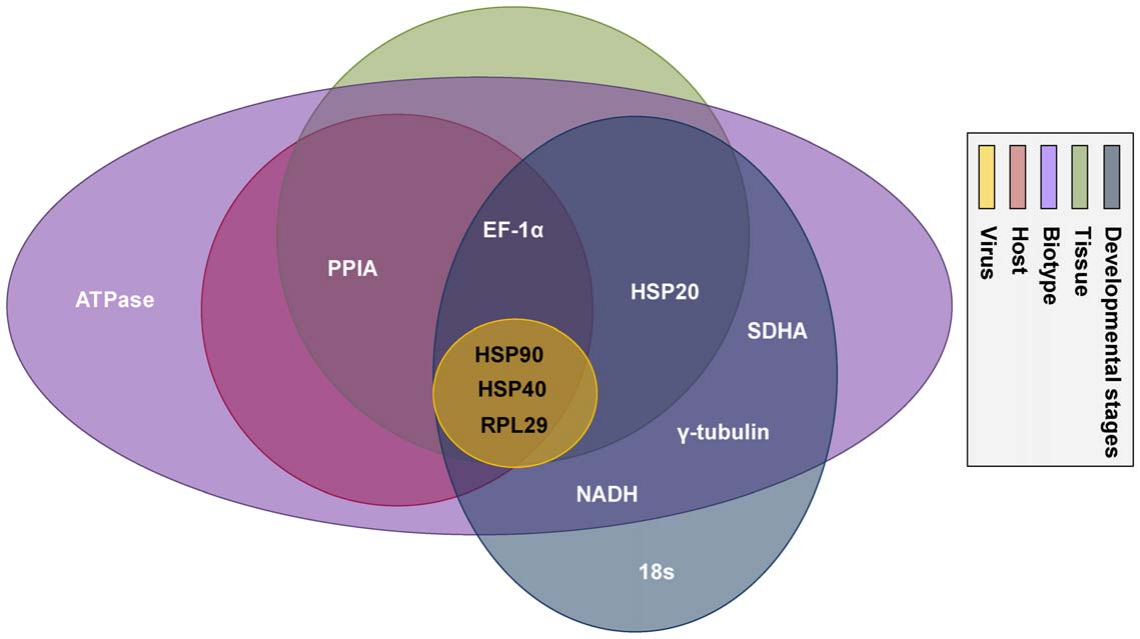
Reference genes selected by *geNorm* under various biotic conditions. The expression stability measure (*M*) is the mean of the stability values of the remaining genes. The least stable genes have the highest *M* values. The genes listed here are considered stable based on a cutoff *M* value of less than 0.5. Each circle with a distinct color represents a different set of biotic condition. Genes located within one circle are stable under a specific biotic condition, and genes shared with multiple circles are stable across those conditions.

For different hosts, reference genes with *M* values<0.5 are ranked (from highest to lowest stability) in the order of *PPIA+EF1-α > HSP90 > HSP40 > RPL29* (Figure 1). For virus status (with or without TYLCV), *RPL29*, *HSP90*, and *HSP40* are the most suited reference genes. For developmental stage, the ranking of reference gene stability among those with *M* values < 0.5 is *HSP90+NADH* > *18S* > *γ-tubulin* > *RPL29* > *EF1-α* > *HSP20* > *HSP40* > *SDHA*. For different *B. tabaci* tissues, *HSP20*, *HSP40*, *HSP90*, *PPIA*, *RPL29*, and *EF1-α* are relatively stable. For whitefly biotype, reference genes with *M* values < 0.5 are ranked (from highest to lowest stability) in the order of *HSP40+NADH* > *SDHA* > *HSP90* > *EF1-α* > *ATPase* > *PPIA* > *γ-tubulin* > *RPL29* > *HSP20*. Based on data obtained with five biotic factors, the ideal reference genes according to *geNorm* are *RPL29*, *HSP40*, and *HSP90*.

**Figure 2:**
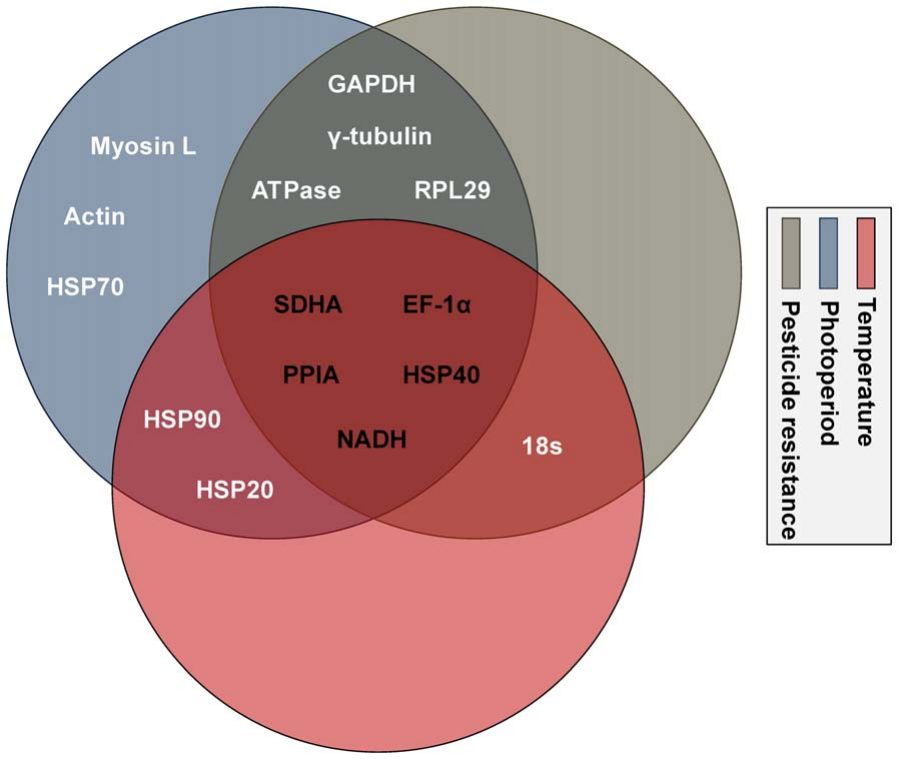
Reference genes selected by *geNorm* under various abiotic conditions. The expression stability measure (*M*) is the mean of the stability values of the remaining genes. The least stable genes have the highest *M* values. The genes listed here are considered stable based on a cutoff *M* value of less than 0.5. Each circle with a distinct color represents a different set of biotic condition. Genes located within one circle are stable under a specific abiotic condition, and genes shared with multiple circles are stable across those conditions.

For photoperiod, the *M* values are <0.5 for all candidate reference genes (Figure 2). For temperature, reference genes with *M* values <0.5 are ranked (from highest to lowest stability) in the order of *EF1-α+NADH* > *SDHA* > *RPL29* > *PPIA* > *HSP40* > *ATPase* > *18S* > *GAPDH* > *γ-tubulin*. For pesticide resistance, reference genes with *M* values<0.5 are ranked (from highest to lowest stability) in the order of *PPIA+NADH*>*HSP20*>*HSP40*>*HSP90*>*18S*>*EF1-α*>*SDHA*. Based on data obtained from three abiotic factors, the ideal reference genes are *EF1-α*, *PPIA*, *NADH*, *SDHA*, and *HSP40*.

#### *Normfinder*

*Normfinder* indicated that *RPL29*, *GAPDH*, and *NADH* are the most stable reference genes for host plants, tissues, biotypes, respectively (Table 2). Specifically, for developmental stages and viruliferous/non-viruliferous *B. tabaci*, *SDHA* is the most stable reference gene. A similar trend is observed under selected abiotic factors, in which *HSP20*, *HSP40*, and *EF1-α* are ranked as the most stable reference genes for photoperiod, temperature, and insecticide susceptibility, respectively (Table 3).

**Table 2:** Ranking of candidate reference genes in response to biotic factors.

Rank	Host		TYLCV		Developmental stages		Tissue		Biotype	
	Gene	SV^1^	Gene	SV	Gene	SV	Gene	SV	Gene	SV
1	*RPL29*	0.197	*SDHA*	0.207	*SDHA*	0.212	*GAPDH*	0.116	*NADH*	0.150
2	*HSP90*	0.318	*RPL29*	0.394	*HSP90*	0.332	*EF-1α*	0.203	*HSP40*	0.172
3	*SDHA*	0.351	*HSP90*	0.394	*EF-1α*	0.359	*RPL29*	0.295	*HSP90*	0.178
4	*NADH*	0.418	*γ-tubulin*	0.457	*RPL29*	0.378	*HSP70*	0.323	*RPL29*	0.263
5	*EF-1α*	0.457	*18s*	0.502	*NADH*	0.393	*ATPase*	0.340	*EF-1α*	0.344
6	*PPIA*	0.491	*HSP40*	0.592	*HSP20*	0.525	*SDHA*	0.363	*ATPase*	0.425
7	*ATPase*	0.568	*PPIA*	0.593	*HSP40*	0.599	*HSP20*	0.559	*HSP20*	0.489
8	*HSP40*	0.604	*NADH*	0.633	*18s*	0.605	*HSP40*	0.675	*γ-tubulin*	0.536
9	*γ-tubulin*	0.638	*HSP20*	0.672	*ATPase*	0.605	*HSP90*	0.807	*PPIA*	0.585
10	*GAPDH*	0.672	*EF-1α*	0.797	*GAPDH*	0.706	*PPIA*	0.858	*Myosin L*	0.812
11	*HSP20*	0.681	*ATPase*	0.875	*PPIA*	0.759	*Actin*	0.979	*18s*	0.841
12	*18s*	0.710	*HSP70*	1.221	*Myosin L*	0.901	*NADH*	1.244	*Actin*	0.945
13	*Actin*	1.186	*Actin*	1.233	*γ-tubulin*	0.936	*18s*	1.318	*GAPDH*	1.125
14	*Myosin L*	1.216	*Myosin L*	1.310	*Actin*	1.101	*Myosin L*	1.512	*SDHA*	1.455
15	*HSP70*	1.240	*GAPDH*	1.587	*HSP70*	1.252	*γ-tubulin*	2.121	*HSP70*	1.563

“1” : Stability Value was evaluated by *Normfinder*.

**Table 3:** Ranking of candidate reference genes in response to abiotic factors.

Rank	Photoperiod		Temperature		Insecticide Susceptibility^1^	
	Gene	SV^2^	Gene	SV	Gene	SV
1	*HSP90*	0.069	*HSP40*	0.263	*EF-1α*	0.162
2	*HSP20*	0.093	*HSP90*	0.353	*ATPase*	0.269
3	*ATPase*	0.162	*EF-1α*	0.375	*SDHA*	0.279
4	*HSP40*	0.181	*PPIA*	0.425	*HSP90*	0.335
5	*RPL29*	0.244	*NADH*	0.429	*PPIA*	0.364
6	*HSP70*	0.262	*SDHA*	0.451	*HSP20*	0.447
7	*EF-1α*	0.292	*RPL29*	0.473	*RPL29*	0.453
8	*SDHA*	0.293	*γ-tubulin*	0.569	*18s*	0.502
9	*NADH*	0.306	*18s*	0.587	*Actin*	0.514
10	*γ-tubulin*	0.341	*ATPase*	0.589	*NADH*	0.590
11	*PPIA*	0.379	*GAPDH*	0.684	*HSP40*	0.607
12	*Actin*	0.385	*Myosin L*	0.917	*γ-tubulin*	0.680
13	*GAPDH*	0.463	*Actin*	1.306	*HSP70*	0.767
14	*Myosin L*	0.605	*HSP20*	1.804	*GAPDH*	1.461
15	*18s*	0.893	*HSP70*	3.981	*Myosin L*	1.484

“1” : Thiamethoxam-resistant and -susceptible whiteflies.

“2” : Stability Value was evaluated by *Normfinder*.

## Discussion

Because it is highly sensitive, specific, accurate, and reproducible, qRT-PCR is, in many ways, superior to conventional methods (northern hybridization and semi-quantitative PCR), and has become an essential tool for gene expression analysis [13], [28], [35]–[39]. qRT-PCR analysis, however, is influenced greatly by the selection of reference genes [10], [40]–[41]. The endogenous reference genes should be stable across different experimental treatments; otherwise, a variable reference gene can compromise the qRT-PCR analysis by introducing artificial changes or masking true changes in target gene expression. Some commonly used reference genes can vary substantially in response to specific experimental conditions [11], [42]–[43]. In this study, we used two Excel-based algorithms *geNorm* and *Normfinder* to evaluate the stability of 15 candidate reference genes in *B. tabaci* in response to five biotic factors (host, virus, stage, tissue, and biotype) and three abiotic factors (photoperiod, temperature, and insecticide susceptibility).

A major conclusion of this study is that many of the candidate genes in *B. tabaci* should not be used as the default reference genes because their expression is highly variable under certain conditions. Our results indicate that the stability of reference gene expression must be validated for each experimental condition under investigation. The ranking of these reference genes differs somewhat for *geNorm* and *Normfinder*, because these programs have different algorithms and different sensitivities toward co-regulated reference genes. Despite the discrepancies, both programs identified a similar set of reference genes suited for the respective experimental conditions.

The ideal reference genes in response to biotic factors were *RPL29*, *HSP40*, and *HSP90* according to *geNorm* and *RPL29* based on *Normfinder*, respectively. Combing these results, *RPL29* is a consensus reference gene that is reliable across a range of biotic conditions (Table 4), and this is consistent with the performance of the other *ribosomal protein L32*, a widely used single normaliser in gene expression studies [11], [43]–[47]. Despite subtle ranking differences between *geNorm* and *Normfinder*, the ideal reference genes in response to abiotic factors were determined to be *EF1-α*, *PPIA*, *NADH*, *SDHA*, and *HSP40* (Table 4). *EF-1α* has rarely been used as a normaliser in the past but has recently been selected as a suitable reference gene in salmon [48], humans [47], [49], Orthoptera [46], [50], and Hymenoptera [43]. *PPIA* was considered sufficiently stable for normalization in this study, which is consistent with a previous report in human cervical tissues [47].

**Table 4:** Recommended reference genes for various experimental conditions.

Experimental Conditions	Recommended Reference Genes		
***Biotic Factors***			
Host	*HSP90*	*RPL29*	*EF-1α*
TYLCV	*HSP90*	*RPL29*	
Developmental stages	*NADH*	*HSP90*	*RPL29*
Tissue	*RPL29*	*EF-1α*	
Biotype	*NADH*	*HSP90*	*EF-1α*
***Abiotic Factors***			
Photoperiod	*HSP40*	*HSP90*	*PPIA*
Temperature	*EF-1α*	*NADH*	*SDHA*
Thiamethoxam susceptibility	*PPIA*	*EF-1α*	*HSP20*

Another conclusion of our study is that some genes that have been consistently used for the normalization study showed high levels of variation in response to certain treatments. Previously, *18S* has been considered an ideal reference gene because the expression level of rRNA appears to vary considerably less than mRNA [51]. In this study, the raw Ct values of *18S* ranged from 9.92 to 15.94 depending on insect body region and host plants, suggesting that the expression of *18S* can be highly variable and consequently, it could not be used as a reference gene under certain experimental conditions. This result is consistent with some earlier studies on *18S RNA* [11], [42]. Another commonly used reference gene, *actin*, encodes a major component of the protein scaffold that supports the cell and determines its shape. The expression of *actin* is moderately abundant in most cell types, and *actin* has been used extensively as a reference gene in *B. tabaci* and in many other insects including the desert locust [46], European honey bee [45], and two species of Collembola [52]. In our study *actin* was not stable among different tissues (body regions) and hosts; disqualifying *actin* as a suitable reference gene under these conditions.

In recent years, more researchers have adopted a multiple reference gene approach to analyze gene expression [38], [53]. Our results demonstrated that the expression of several reference genes from *B. tabaci* were consistently stable across selected experimental conditions. However, the best-suited reference genes can be different in response to diverse biotic and abiotic factors (Table 4). Our finding is the very first step toward establishing a standardized qRT-PCR procedure following the MIQE (Minimal Information required for Publication of Quantitative Real-Time PCR) guideline in an agriculturally important insect pest. More importantly, this study provides a solid foundation for future RNAi-based functional study in *B. tabaci*.

## Supporting Information

Figure S1 **Optimal number of reference genes required for accurate normalization of gene expression under biotic conditions.** Based on *geNorm* analysis, average pairwise variations are calculated between the normalization factors NF_n_ and NF_n+1_ to indicate whether inclusion of an extra reference gene increases the stability of the normalization factor. Values<0.15 indicate that additional genes are not required for the normalization of gene expression.
(TIFF)

Figure S2 **Optimal number of reference genes required for accurate normalization of gene expression under abiotic conditions.** Based on *geNorm* analysis, average pairwise variations are calculated between the normalization factors NF_n_ and NF_n+1_ to indicate whether inclusion of an extra reference gene adds to the stability of the normalization factor. Values<0.15 indicate that additional genes are not required for the normalization of gene expression.
(TIFF)
