# Supplementary figures and images for: Reference Gene Selection for qRT-PCR Analysis in the Sweetpotato Whitefly, Bemisia tabaci (Hemiptera: Aleyrodidae)
Source: PLoS One. 2013 Jan 8;8(1):e53006. doi: 10.1371/journal.pone.0053006 (PMC3540095; doi:10.1371/journal.pone.0053006)

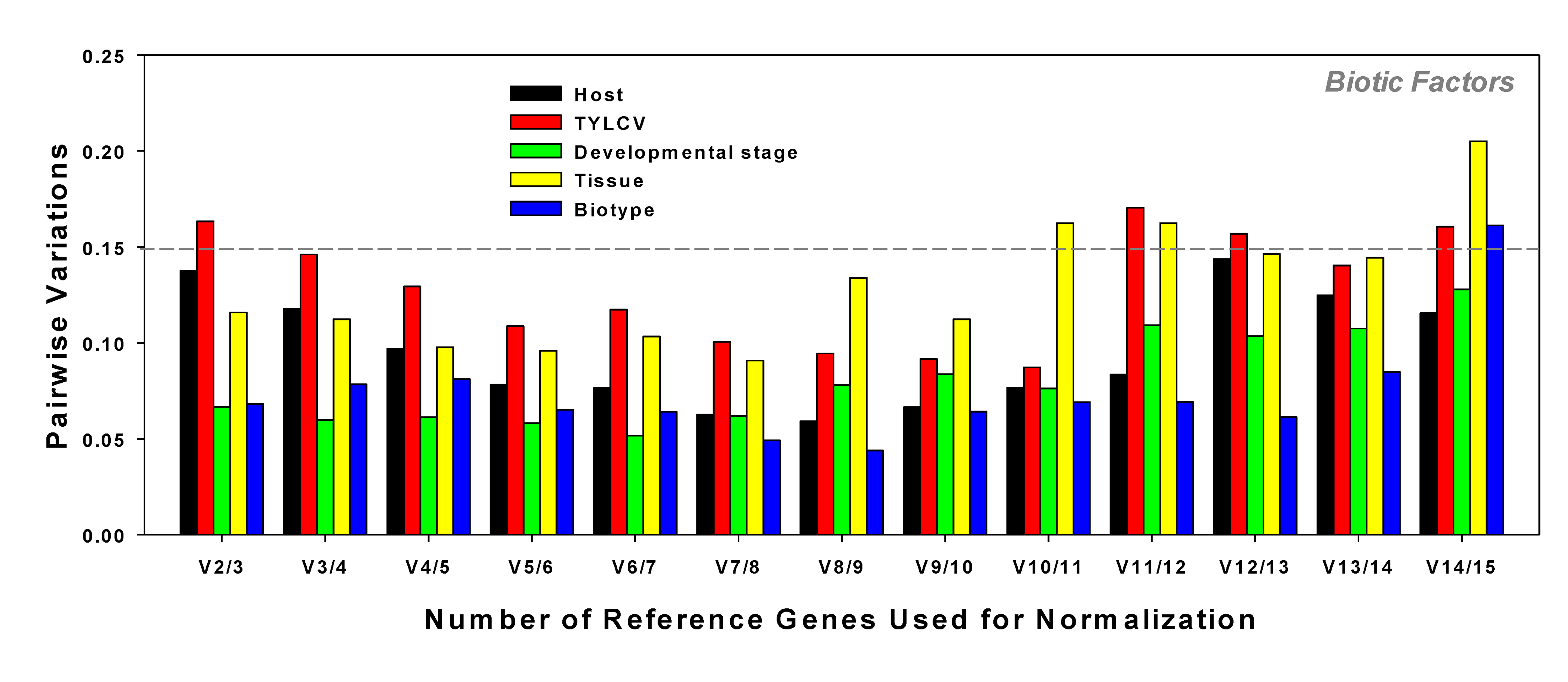

Supplement: Figure S1 — Optimal number of reference genes required for accurate normalization of gene expression under biotic conditions. Based on geNorm analysis, average pairwise variations are calculated between the normalization factors NFn and NFn+1 to indicate whether inclusion of an extra reference gene increases the stability of the normalization factor. Values<0.15 indicate that additional genes are not required for the normalization of gene expression. (TIFF) [file pone.0053006.s001.tiff]

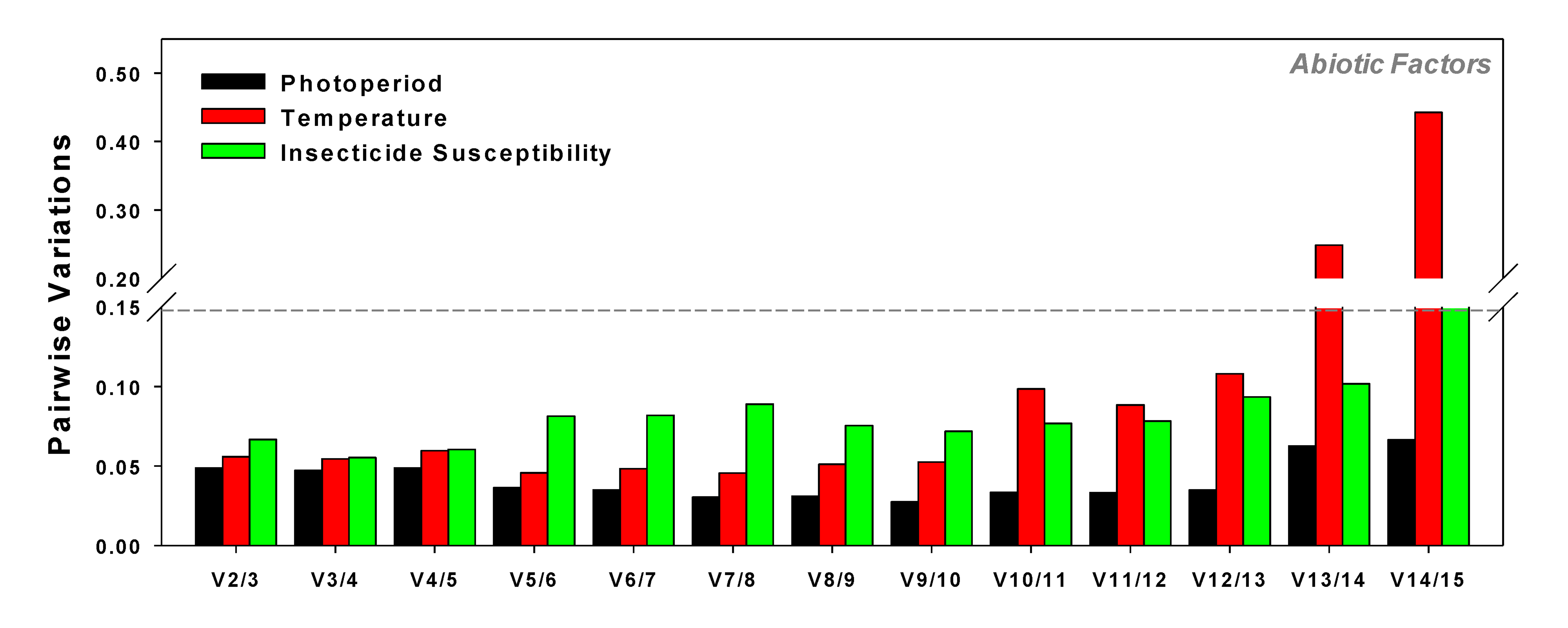

Supplement: Figure S2 — Optimal number of reference genes required for accurate normalization of gene expression under abiotic conditions. Based on geNorm analysis, average pairwise variations are calculated between the normalization factors NFn and NFn+1 to indicate whether inclusion of an extra reference gene adds to the stability of the normalization factor. Values<0.15 indicate that additional genes are not required for the normalization of gene expression. (TIFF) [file pone.0053006.s002.tiff]
